# “IS IT TIME TO STOP DRIVING?”: A RANDOMIZED TRIAL OF AN ONLINE DECISION AID FOR OLDER DRIVERS

**DOI:** 10.1093/geroni/igac059.2285

**Published:** 2022-12-20

**Authors:** Faris Omeragic, Lauren Meador, Nicole Fowler, Rachel Johnson, Elizabeth Boland, Ryan Peterson, Marian Betz

**Affiliations:** University of Colorado School of Medicine, Aurora, Colorado, United States; Stanford University School of Medicine, Stanford, California, United States; Indiana University School of Medicine, Indianapolis, Indiana, United States; Colorado School of Public Health, Aurora, Colorado, United States; University of Colorado Anschutz Medical Campus, Aurora, Colorado, United States; University of Colorado, Aurora, Colorado, United States; University of Colorado School of Medicine, Aurora, Colorado, United States

## Abstract

The decision to stop or continue driving can be challenging for older adults. In a prospective two-arm randomized trial, we sought to test whether an online driving decision aid (DDA) would improve decision quality. We recruited 301 English-speaking licensed drivers, age ≥70 years, without significant cognitive impairment but with ≥1 diagnosis associated with increased likelihood of driving cessation, from clinics associated with study sites in three states. They were randomized to view 1) the online Healthwise® DDA for older adults addressing “Is it time to stop driving?”; or 2) a control condition of web-based information. Our primary outcome was decision conflict as estimated by the Decisional Conflict Scale (DCS; lower scores indicate higher quality). Secondary outcomes were knowledge and decision self-efficacy about driving decisions. We examined differences in post-randomization outcomes by study arm using generalized linear mixed-effects models with adjustment for site and pre-randomization scores. Intervention participants had a lower mean DCS score (12.3 DDA vs 15.2 control; p=0.017) and a higher mean knowledge score (88.9 DDA vs 79.9 control; p=0.038); we found no difference between groups in self-efficacy scores. The DDA had high acceptability; 86.9% of those who viewed it said they would recommend it to others in similar situations.The online Healthwise® DDA decreased decision conflict and increased knowledge in this sample of English-speaking, older adults without significant cognitive impairment. Use of such resources in clinical or community settings may support older adults as they transition from driving to other forms of mobility.

